# A103 GUTLINK SMARTPATH: AN ONLINE TOOL TO IMPROVE PATIENT CARE

**DOI:** 10.1093/jcag/gwae059.103

**Published:** 2025-02-10

**Authors:** G Park, M Stewart, J Jones, N Willett

**Affiliations:** Dalhousie University, Halifax, NS, Canada; Dalhousie University, Halifax, NS, Canada; Dalhousie University, Halifax, NS, Canada; Dalhousie University, Halifax, NS, Canada

## Abstract

**Background:**

Lower gastrointestinal (GI) symptoms are common reasons patients present to primary healthcare providers (PHCP). It can be challenging for PHCPs to identify dietary and functional conditions versus serious illnesses that require specialty assessment. Unfortunately, access to GI care in Nova Scotia is limited with patients waiting well beyond published standards.

The Division of Digestive Care and Endoscopy (DDCE) at Dalhousie University has developed a referral management system to prioritize patients. Unfortunately, the lack of standardization in referral requirements creates challenges to implementing a robust and accurate referral management system.

To support the evaluation and management of patients with lower GI symptoms within primary care, we developed an adaptive clinical care pathway (SmartPath), delivered within an online platform, Virtual Hallway (VH). The SmartPath is an evidence-informed clinical support tool facilitating appropriate primary care investigations and management as well as specialist referrals, as required.

**Aims:**

To evaluate the implementation and early effectiveness of the lower GI symptom SmartPath.

**Methods:**

We conducted a cohort study of SmarthPaths initiated with primary care. Descriptive data was extracted from the Virtual Hallway platform, with results of a short survey PHCPs were asked to complete following completion of a SmartPath. To assess the quality of referrals facilited by SmartPath versus traditional referral pathways, a series of 20 consecutive referrals for 3 common lower GI complaints (diarrhea, constipation and rectal bleeding) were selected for comparison. Interim data is presented for Smartpaths initiated between October 2023 and August 2024.

**Results:**

A total of 272 SmartPaths were initiated by 103 unique PCHPs, of which 179 (65.8%) led to a specific action, with the 93 (34.2%) remaining in process within primary care. Of the 37 DDCE referrals, 26 (62.1%) were accepted, 11 (29.7%) were declined or redirected to another service.

60 consecutive traditional lower GI symptoms referrals were analyzed, 27 (45%) of which were declined. 31 (51.7%) did not provide any investigations, 42 (70%) did not provide physical examinations, and 34 (56.7%) did not provide a past medical history.

Survey responses were provided by 57 PHCPs with mean scores (out of 5) of 4.2 for ease of use, 3.9 for overall satisfaction, and 4.2 for likelihood to use again.

**Conclusions:**

An adaptive care pathway is effective for facilitaing investigation and management of common lower GI symptoms within primary care while facilitating access to specialized services, as required. Referrals received through smartpath had a higher acceptance rate.

**Funding Agencies:**

**None**

